# Better together - efficacy of combined physical and cognitive training in young adults for enhancing physical and cognitive performance: a randomized controlled trial

**DOI:** 10.3389/fspor.2026.1842483

**Published:** 2026-06-24

**Authors:** Amanda Scott, Keely McDicken, Kristy Martin, Joseph M. Northey, Amit Lampit, Andrew Flood, Richard Keegan, Alison Fogarty, Philip Temby, Matthew Driller, Ben Rattray

**Affiliations:** 1 Faculty of Health, UC Research Institute of Sport and Exercise, University of Canberra, Canberra, ACT, Australia; 2 Discipline of Sport and Exercise Science, Faculty of Health, University of Canberra, Canberra, ACT, Australia; 3 Department of Psychiatry, University of Melbourne, Carlton, VIC, Australia; 4 Faculty of Heath, Southern Cross University, Lismore NSW, Australia; 5 Department of Defence, Defence Science and Technology Group, Adelaide, SA, Australia; 6 School of Allied Health, Human Services, and Sport, La Trobe University, Melbourne, VIC, Australia

**Keywords:** cognition, concurrent training, dual-tasking, memory, reaction time, simultaneous

## Abstract

**Background:**

Young adults face heightened cognitive demands from modern work and societal pressures, necessitating interventions to support cognitive performance. Research on concurrent training, whereby physical and cognitive training are performed simultaneously, suggests benefits for enhancing physical and cognitive performance in a time-efficient manner. This study aimed to evaluate the effect of concurrent training on physical and cognitive performance in young, healthy adults compared to separate or physical-only training.

**Methods:**

A 12-week randomized controlled trial was conducted with 107 participants (25 ± 6 yrs, 58 females) allocated to one of three groups: 1) bi-weekly stationary cycling (physical); 2) bi-weekly physical training while performing simultaneous cognitive training (concurrent); or 3) bi-weekly physical training plus bi-weekly cognitive training (separate). Physical training involved stationary cycling at 70%–80% of maximal heart rate achieved during baseline testing. Cognitive training was delivered using an adaptive, multi-domain computer program. Physical and cognitive performance was assessed before and after the 12-week intervention via a cognitive battery and maximal cardiorespiratory fitness test. To assess differences in intervention efficacy, all participants randomized with baseline assessments were included in intention-to-treat analysis using linear mixed effects models.

**Results:**

Across all interventions, response speed improved in tasks requiring rapid executive function including Visual Search, Stroop, and Category Switch. However, the concurrent and physical training groups achieved this improvement with comparatively smaller declines in accuracy. Reaction time in the Psychomotor Vigilance Task increased across groups, with the physical group showing slower reaction time and more lapses than the concurrent and separate training groups. Cardiorespiratory fitness improved most for the separate group, but, despite this, time-to-exhaustion improved in all groups with no difference between interventions.

**Conclusion:**

The results of this study demonstrate improvement to components of cognitive performance in young adults over a 12-week intervention. Reaction time improved across various cognitive tasks for all groups, however, the concurrent group demonstrated this improvement most consistently, and with less compromises to accuracy. Despite the separate group appearing favourable for cardiorespiratory fitness, there were no differences for time-to-exhaustion. Given the time-efficient nature of concurrent training, this modality shows promise for developing both physical and cognitive performance in young healthy adults, relative to other active interventions.

## 1 Introduction

Modern occupational environments, study demands, and societal expectations place increasing cognitive demands on young adults (1). Therefore, young adults may benefit from interventions which aim to improve cognitive performance, equipping them to meet these challenges effectively. Such improvements to cognitive performance may support individual self-efficacy (2), quality of life (3), and a greater cognitive reserve for later life (4). To address these challenges, evidence-based interventions that support or enhance cognitive performance are needed, with physical activity and cognitive training representing two widely studied approaches aimed at brain health and cognitive function.

Physical activity guidelines recommend regular moderate-intensity aerobic activity for overall physical health (5), and increasingly, its potential to promote brain health and cognitive function across the lifespan (6). Moderate-intensity aerobic exercise has been shown to produce several neural and vascular adaptations including an increase in cerebral blood flow (7) and circulating levels of brain-derived neurotrophic factor (BDNF) a protein that supports a neural environment conducive to learning, memory and overall cognitive function (8, 9). Despite these recommendations and potential mechanistic pathways, few physical activity interventions targeting cognition in younger adults exist (8, 10–13). Research investigating the effects of physical activity in young adults report benefits to cognitive performance, including executive function (8, 10, 13), processing speed (11), and memory (8, 12), although these effects are typically small (8, 10, 13). This research, however, examines physical activity in isolation and the extent to which physiological adaptations from physical exercise translate to meaningful cognitive benefits remains an area of ongoing scientific debate (14, 15). To contribute to this ongoing discussion and strengthen understanding, research should explore how physical activity can work as an adjunct treatment, supporting cognitive maintenance and enhancement strategies (6, 16, 17).

There is evidence that environments enriched with cognitive stimuli may improve performance across various cognitive domains (18–20). This has likely contributed to the growing popularity of cognitive training, particularly through the promotion of commercial technologies. However, despite its popularity, the limited available evidence suggests that cognitive training yields only small improvements in healthy young adults (21), and that these effects appear reliant on tasks being sufficiently demanding (22) and tailored to the individual (20). Given moderate-intensity exercise has been linked to supporting a neuroplastic environment, a combined approach may offer an opportunity to augment cognitive specific training, forming the theoretical rational underpinning the present study design.

While there are countless methods to combine physical and cognitive training, the emerging literature typically groups these methods into separate (each stimulus in discrete sessions) (23), sequential (one stimulus follows the other within the same session) (24) or concurrent (both stimuli delivered simultaneously) (24). In older adults, the combination of physical and cognitive training with concurrent methods has been shown to provide more efficacious improvements than separate or sequential methods (24), as well as reducing the time burden of training. Available evidence suggests a combined dose of 1–3 h a week for 12–16 weeks (24), and although these results are promising, the efficacy and acceptability of concurrent physical and cognitive training interventions in young adults remains unclear.

Some initial research has been conducted in younger, healthy populations; however, this work has often been driven by alternative mechanistic rationales (e.g., improved glucose metabolism with concurrent training) (25) or focused primarily on resilience to mental fatigue (26), or physical performance outcomes (27–32). Furthermore, this early research has often been limited by underpowered study designs and a lack of focus on targeted, adaptive cognitive training, which may have constrained the cognitive benefits observed.

Therefore, given the limited evidence and methodological variability within concurrent training research in young adults, the present study employed a randomized controlled trial (RCT) comparing the effects of physical training performed either alone, concurrently with cognitive training, or in a separate session to cognitive training. In acknowledging the physical activity requirements of a healthy population, we implemented a dedicated physical activity control group to provide a relevant comparison. As such, the primary aim of this study was to investigate the efficacy of the two combined physical and cognitive training interventions on cognitive performance outcomes, compared to physical only training over the 12-week intervention period. A secondary aim was to evaluate the impact of the interventions on physical performance outcomes.

## 2 Materials and methods

### 2.1 Design

**Figure 1:**
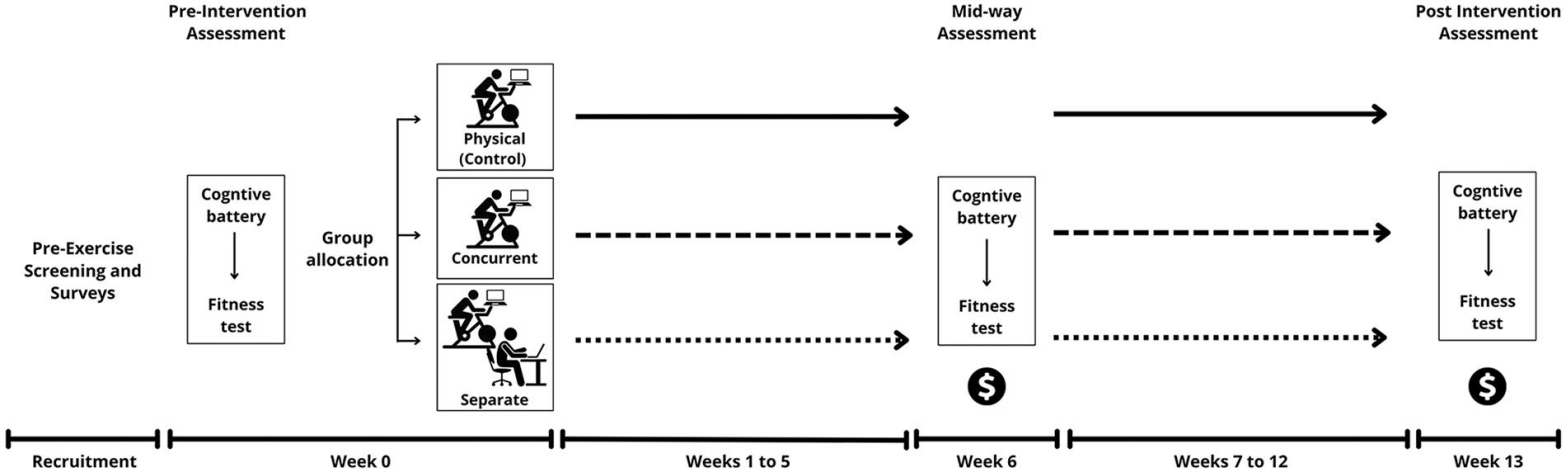
Schematic depicting experimental protocol; participants underwent pre-exercise screening before pre-intervention assessments which consisted of a cognitive battery followed by a maximal incremental fitness test. Participants were then randomly allocated to a group before commencing the intervention. Financial reimbursement was provided in week 6 and week 13 once assessments were completed.

A 12-week, three-arm, investigator-blinded RCT was conducted across two collection sites at the University of Canberra and La Trobe University. After completing pre-exercise screening and profiling surveys, participants attended an information session covering the interventions, baseline testing, and a short visual demonstration of the cognitive assessments. Primary assessments were conducted pre-intervention (baseline) and post-intervention (within 1 week; see Figure 1). These assessments consisted of a cognitive battery followed by a maximal incremental fitness assessment. Participants were instructed to abstain from alcohol the night before, and caffeine the day of each assessment, as well as consume their last meal at least one hour before the fitness test. Financial reimbursement was provided to participants once assessments were completed. Participants provided written informed consent and the project was approved by the University of Canberra Human Research Ethics Committee (HREC10431).

### 2.2 Participants

**Figure 2:**
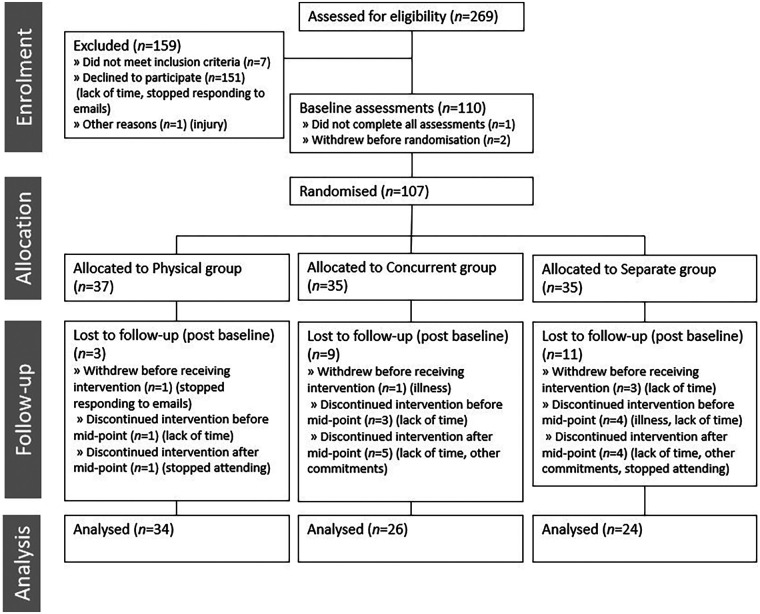
Flow of participants through interventions by group; following recruitment, screening, and pre-intervention assessments, a total of 107 participants were randomized to the three intervention arms. Scheduling conflicts and injury/illness were the primary cause of attrition with most withdrawals (*n* = 11) occurring in the separate group.

Participants (*n* = 107 [f = 58]; mean age=25 ± 6 yrs; baseline V̇O_2peak_ 36.8 ± 9.2 mL·kg^−1^·min^−1^) (see Table 1) were recruited across two university campuses, through online faculty bulletin boards, posters, email lists, and social media between May 2022 and May 2023. Males and females were included if they were aged between 18 and 40 years (inclusive), self-reported recreationally active or trained (33) and passed pre-exercise screening (34). Participants were excluded if they declared any medical condition, injury, or medications that prohibited them from completing any of the training interventions, or that would impact the outcome measures (e.g., color blindness or taking psychoactive medications). Participants were invited to the laboratory on two occasions to complete pre-intervention assessments and surveys. Upon completing assessments, participants were admitted into the study and randomized to an intervention group (Figure 2).

**Table 1:** Baseline characteristics of participants after randomization V̇).

Group	Age (yrs)	Female, *n* (%)	Height (cm)	Body Mass (kg)	V̇O₂peak (mL·kg⁻^1^·min⁻^1^)	BMI (kg/m^2^)
Physical	24 ± 6	20 (54%)	171.7 ± 9.9	72.0 ± 16.8	36.4 ± 8.4	24.2 ± 4.1
Concurrent	24 ± 6	19 (54%)	171.4 ± 8.4	67.9 ± 11.3	38.7 ± 10.6	23.0 ± 2.6
Separate	26 ± 5	19 (54%)	169.2 ± 9.1	71.4 ± 13.6	35.4 ± 8.2	24.9 ± 4.4

Data presented as mean ± SD unless otherwise indicated. No differences were observed between groups (*p* > 0.05). SD, Standard Deviation.

#### 2.2.1 Sample size

A power analysis was completed a-priori using G*power (35) to determine the minimum sample size for a three-group, repeated measures randomized controlled trial. The analysis suggested a sample size of 84 (28 per group) would be required to detect an effect size of d = 0.35 (f = 0.175), with an *α* of 0.05% and 80% power. The effect size was based on a prior meta-analysis (24) and commentary regarding existing studies lacking efficacious training principles. In the absence of existing research in young, healthy populations, an effect size from a prior meta-analysis in older adults was considered appropriate to provide an evidence-based estimate, although we acknowledge potential differences between populations. We estimated a 15% dropout rate across groups leading to a recruitment target of 107 participants.

#### 2.2.2 Randomization, group allocation and blinding

An online, computer-generated, random sequence of numbers in blocks of variable sizes (3, 6, 9) in a 1:1:1 ratio for the three experimental groups stratified by sex (female and male) and site (Canberra and Melbourne) was generated by a researcher external to the study. After pre-intervention testing, group allocations were handed to participants in a sealed, opaque envelope. Based on the nature of the study, participants and staff involved in delivering the intervention were not blinded to group allocation. However, all assessments were carried out by blinded assessors.

### 2.3 Interventions

#### 2.3.1 Physical training (physical)

**Figure 3:**
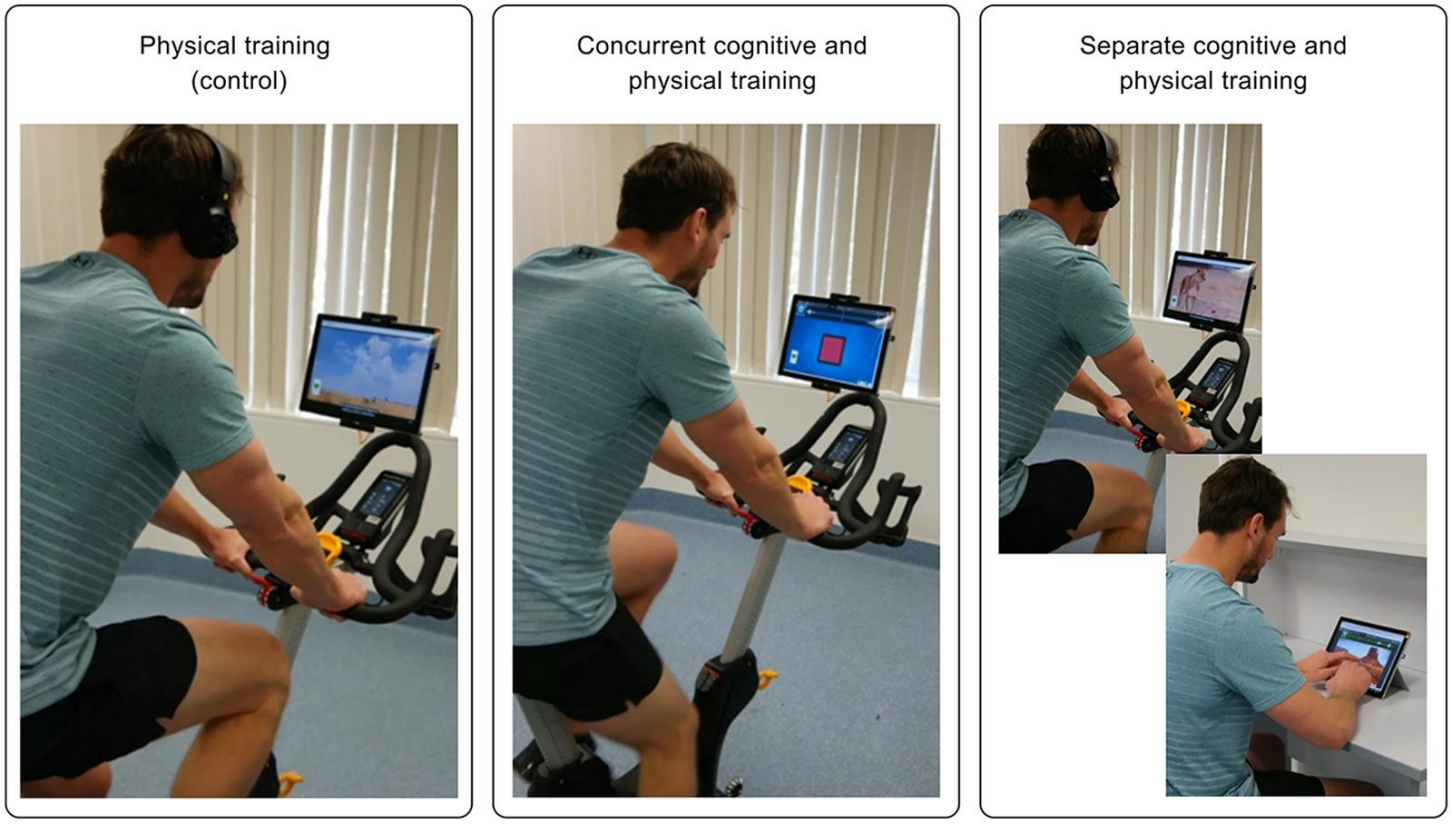
Visual depiction of intervention groups; physical training where participants would engage in moderate-intensity exercise on a stationary bike whilst simultaneously watching videos on YouTube; concurrent training which required simultaneous engagement in physical training (the same as the physical group) and cognitive training on a mounted tablet; and separate training where participants conducted the same physical training as the physical group and attended additional sessions to conduct cognitive training seated, and at rest.

Participants attended two one-hour sessions each week, for 12 consecutive weeks. Physical training was conducted on a cycle ergometer (CXP Target Training Cycle, Matrix Fitness, Dandenong South, Australia) for a total of 56 min consisting of a 3-minute warm-up (30%–65% of HRpeak achieved during baseline testing), 50 min of moderate exercise (70%–80% of HRpeak) and a 3-minute cooldown (30%–65% of HRpeak). During the 50 min of moderate exercise, participants simultaneously watched videos from a dedicated YouTube channel curated with engaging, yet emotionally neutral content (Figure 3). The inclusion of the videos sought to provide a passive, low-demand viewing condition, mirroring the visual attention requirements of the concurrent training intervention, but with little higher-order cognition.

#### 2.3.2 Concurrent physical and cognitive training (concurrent)

Participants in the concurrent condition also attended two one-hour training sessions each week, for 12 weeks. The physical training component was the same as the physical group, however, instead of watching emotionally neutral videos, participants simultaneously completed computerized cognitive training via BrainHQ (Posit Science, California, USA).

BrainHQ is an adaptive, multi-domain web-based program which applies progressive overload principles by adjusting task difficulty to user performance. The BrainHQ platform has been used previously to successfully enhance cognitive performance in older adults (36, 37).

Throughout cognitive training, eight tasks were employed to provide variation, novelty, and an appropriate challenge to the cognitive domains assessed in the cognitive battery pre- and post-intervention. Further details of the cognitive training are provided in McDicken et al. (38).

#### 2.3.3 Separate physical and cognitive training (separate)

Participants in the separate training group attended two physical training sessions and two cognitive training sessions each week, for 12 weeks. The physical sessions were carried out as per the physical intervention. During the cognitive sessions, participants completed the same 50-minute bout of BrainHQ training as the concurrent intervention, but whilst seated and at rest.

### 2.4 Measures

#### 2.4.1 Adherence

Adherence to the intervention was evaluated using attendance and withdrawal rates. Attendance was measured for all participants by recording the number of training sessions attended. Participants who elected to end their participation in the study or did not return for post-intervention assessments were treated as a withdrawal. Participants that withdrew from the study were not included in the attendance data however, the date of withdrawal was recorded and feedback regarding reasons for withdrawal requested. Participants were required to attend at least 80% of sessions to receive financial reimbursement.

#### 2.4.2 Cognitive performance

##### 2.4.2.1 Cognitive battery

The computerized cognitive assessment, consisting of six tasks completed in the order described below, took ∼35 min to complete and was conducted in a quiet room. All tasks were downloaded on 6th April 2022 from the Millisecond (Seattle, USA) test library and delivered using the Inquisit Player version 6.5.2 in English. Tasks were selected for their relevance to assessing executive function, vigilance and decision-making which are critical in demanding occupations such as emergency services, military, and professional sporting contexts. Each assessment included practice trials before the test trials. Minor modifications were made to either the duration (i.e., the number of practice or test trials) or the content of the tasks (i.e., choice of words to eliminate colloquial language). A detailed description of the procedures and modifications for each task can be found in Appendix A, with a brief overview of each task provided below.

###### 2.4.2.1.1 Digit Span

The Auditory Digit Span (39) script was used to assess verbal short-term memory by audibly presenting digit-sequences to recall. Participants listened to digit-sequences, then used a mouse to select the numbers in the correct sequence on the screen. The task consisted of 14 trials, starting with three digits, and the number of digits in the sequence was adjusted based on the participants’ performance. Following the forward span task, participants completed the backward span task, requiring recall in reverse order. Overall, the assessment lasted four minutes. Two-error maximum length (memory) was recorded.

###### 2.4.2.1.2 Visual Search Task

An adapted version of the Inquisit Visual Search task (Divided Attention) (40) was utilized to assess visuo-spatial working memory. Participants were required to identify target stimuli amidst varying numbers (6, 12, 24, or 48) of distractors. Each trial began with a fixation followed by a brief display of the target stimuli, after which an array of distractor elements appeared. Participants were required to identify the presence or absence of the target and respond accordingly. The visual search task was reduced from 192 to 48 trials (∼5 min) with the array size and target characteristics randomized. Mean reaction time for correct responses and overall accuracy was recorded.

###### 2.4.2.1.3 Stroop

The Color Word Stroop (41) with keyboard responding task was used to assess response inhibition. Four words (‘blue’, ‘green’, ‘red’, and ‘black’) were sequentially displayed in the center of the screen in varying colors (blue, green, red, or black) that may or may not have corresponded to the meaning of the word. Participants were instructed to identify the color of the word, rather than its meaning. The number of trials was increased from 84 to 108 to match the duration of other tasks. Trials were divided evenly between congruent (the text color matched the word's meaning), incongruent (the text color did not match the word's meaning) and control trials (colored rectangles). Overall mean reaction time and accuracy were recorded.

###### 2.4.2.1.4 Category Switch Task

The Category Switch task (42) was included to assess attention-switching performance. This task involved the categorization of words into four groups based on size and living/non-living status. Participants responded to each word by determining its category using a displayed symbol above the word and pressing corresponding keyboard keys. The trials involved switching between congruent (correct responses for both categories require the same button to be pressed) and incongruent (correct responses for both categories require opposite buttons to be pressed) or vice versa. The task included 32 trials lasting approximately five minutes. The original script was adapted to limit ambiguity by changing some words (see Appendix A for detail). Reaction time and accuracy for both switch and non-switch trials were recorded.

###### 2.4.2.1.5 Millisecond Gambling Task

Decision-making and risk-taking were assessed using the Millisecond Gambling Task; an adaptation of the Cambridge Gambling Task (43). In this task, participants choose between red and blue boxes, aiming to find a hidden yellow token. The task comprises three stages: practice, a series of nine ascending bets, and nine descending bets. In the betting rounds, participants wager points based on their color selection, with bet increments increasing or decreasing at intervals representative of 5%, 25%, 50%, 75% and 95% of current points. The number of betting trials was reduced to 18 to restrict the assessment to 4 min. Each bet was displayed on the screen for 2000ms, reduced from the default of 5000 ms. The percentage of best choice metric was recorded.

###### 2.4.2.1.6 Psychomotor Vigilance Task

The Psychomotor Vigilance Task (PVT) was included as a test of sustained attention and simple motor reaction time (44). In this task participants are instructed to press a response key immediately upon seeing a red stopwatch appear on the screen. The test duration was reduced from 10 to 5 min, consistent with shorter versions that have been validated (45). Mean reaction time and number of lapses (46) were recorded.

#### 2.4.3 Subjective workload

##### 2.4.3.1 NASA task load Index

Participants rated the subjective workload of the cognitive battery using the NASA-Task Load Index (NASA-TLX) (47). The NASA-TLX uses six subscales to quantify overall workload, five of which were used in the present study: mental demand, physical demand, temporal demand, effort, and frustration. Participants were required to score each of the subscales on a scale divided into 20 equal intervals anchored by the descriptors of very high and very low. This score was multiplied by five, resulting in a final score between 0 and 100 for each of the subscales.

#### 2.4.4 Physical performance

##### 2.4.4.1 Maximal fitness test

After completing the cognitive battery and NASA-TLX, participants completed a maximal incremental fitness test. Each participant had their height and weight recorded and was fitted with a heart rate monitor (Polar H10). The test was completed on an upright stationary cycle ergometer (Lode Corvial CPET, Groningen, Netherlands), with respiratory data collected via a metabolic cart (Canberra: Vyntus CPX, Vyaire Medical, America; Melbourne: Parvo TrueOne 2400, Parvo Medics, Utah, USA). It was reiterated that participants needed to continue for as long as they could to ensure an accurate measure of fitness was obtained. The protocol included a three-minute warm up at 50 W followed by an incremental assessment period where the resistance on the bike increased every two minutes by 25 W (females) or 50 W (males). Participants were required to pedal at a cadence between 60 and 80 revolutions per minute (RPM). Real-time visual feedback using a screen mounted to the bike and verbal prompting were used to maintain cadence. Heart rate (HR) was recorded via a Polar H10 monitor every 15 s and the rating of perceived exertion (RPE) was taken at the end of each stage using the Borg's 6–20 scale (48). The test was terminated when the participant voluntarily stopped, or their RPM fell below 60 and they were unable to increase it within five seconds. Physiological responses were recorded including breath-by-breath peak oxygen consumption (V̇O_2peak_) and HR using a 15 s average. Relative V̇O_2peak_ was recorded along with the time-to-exhaustion.

### 2.5 Data analysis

All participants randomized into an intervention group (having completed pre-intervention assessment) were included in an intention-to-treat analyses. Means and standard deviations were calculated for attendance with group differences investigated with linear models. Differences in group dropout rates were assessed using Fisher's exact test and pairwise comparisons. To assess the primary and secondary aims, the differences in intervention efficacy, linear-mixed effects models were used to compare performance with the interaction between group and time as the fixed effect, and participant included as a random effect. Where interaction effects were non-significant, the interaction term was dropped, and both group and time were included as fixed effects. Effect sizes were calculated using *post hoc* contrasts from the linear-mixed models. Where interactions in pre- to post-intervention time points were present, we were interested in the time course of intervention effects on the variables of concern. Consequently, we additionally ran linear-mixed effect models using the pre- to mid-intervention, and mid- to post-intervention timepoints. Linear mixed models provide an appropriate analysis given they can provide unbiased estimates (49) through utilizing all available data, including participants with missing data, and account for the repeated measures nature of the data and interindividual variability through the random intercept fitted for participants. This approach appears robust when differential participant attrition is present across intervention arms (50, 51). Assumptions regarding the models were evaluated by visually inspecting the residuals as a QQ plot and scatterplots of the residuals grouped by group and time.

All data are reported as mean and standard deviation. The study accepted an a-priori alpha of 0.05 to determine significance and report effect sizes and confidence limits where appropriate. All analyses were conducted in R (52) (R version 4.3.1) using the lme4 (53), lqmm, and emmeans (54) packages.

## 3 Results

Full data are presented within the Appendix for each outcome variable. Further information on the physical and cognitive load elicited by the interventions is reported in McDicken et al. (38). Where significant interactions were observed from pre- to post-intervention, further exploration of the results using the mid-assessment is included in the Appendix.

### 3.1 Adherence

Following randomization, 23 participants (21%) elected to withdraw from the study (Figure 2). There was a significant effect for group (*p* = 0.036), with the separate group having a higher withdrawal rate (*n* = 11) than the physical group (*n* = 3, *p* = 0.046). No other differences were observed between groups (concurrent: *n* = 9, *p* > 0.122). The most common reason for withdrawing (*n* = 18 of 23) was due to a change in availability or scheduling conflicts (i.e., the time requirement for participation) (see Appendix B).

Attendance was measured as a percentage of total prescribed sessions. Accounting for the mid-assessment interruption, the total number of sessions was 23 for the physical and concurrent groups and 46 sessions for the separate group (comprising 23 physical and 23 cognitive sessions). Participants were expected to complete all scheduled sessions, and any missed sessions were rescheduled where possible, with a minimum of 24 h between consecutive sessions.

Overall attendance for the physical training sessions was 94 ± 7% with no group differences (concurrent: 92 ± 7%; separate:96 ± 6%, physical: 93 ± 7%, *p* = 0.220). The separate group had a mean attendance rate of 96 ± 4% for cognitive training sessions (range:83%–100%).

Four participants reported effects related to increased leg fatigue during the intervention; however, these were not classified as adverse events due to their mild nature, transience, and the expected physiological response in this type of physical training. No other adverse events were reported.

### 3.2 Impact of interventions on cognitive performance

#### 3.2.1 Digit span

There were no interactions between group and time for digit span variables (forwards: F_2,90_ = 0.432, *p* = 0.650; backwards: F_2,85_ = 0.39, *p* = 0.678). However, there were time effects (forward: F_1,91_ = 10.43, *p* = 0.002; backward: F_1,86_ = 31.77, *p* < 0.001), with improvements across all groups for both forward (*b* = 0.5 digits) and backward span memory (*b* = 0.8 digits) from pre- to post-intervention.

#### 3.2.2 Visual search task

**Figure 4:**
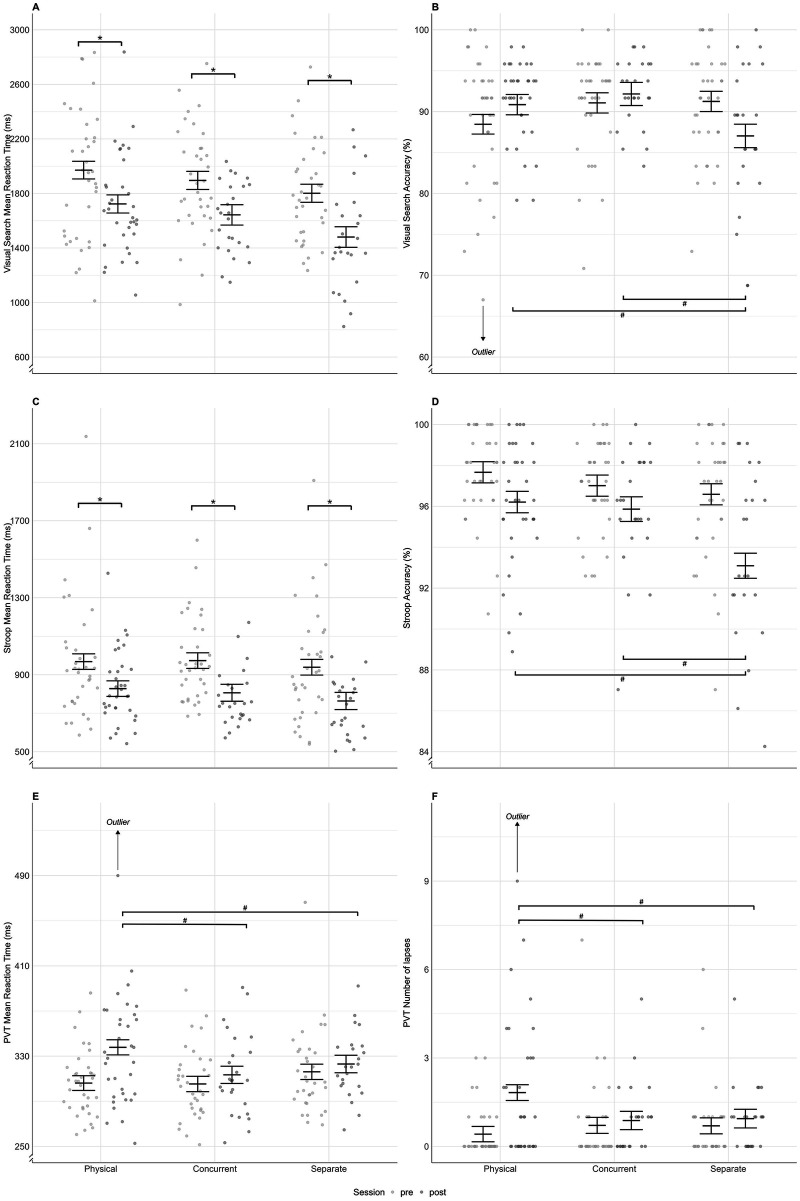
Cognitive performance outcomes from pre- to post-intervention are presented as boxplots. Mean reaction time for the Visual Search (panel **A**), Stroop **(C)**, and Psychomotor Vigilance Task (PVT, panel **E**). Panels **B** and **D** show corresponding accuracy measures. Panel **F** presents the number of lapses recorded during the PVT. Significant main effects for time (*) and group by time interactions (^#^) are denoted with all effects significant at p < 0.05.

There was no interaction between group and time for overall mean reaction time in the visual search task (F_2,88_ = 0.369, *p* = 0.69). There was, however, a main effect for time (F_1,89_ = 52.551, *p* < 0.001), with all groups improving from pre- to post-intervention (faster reaction times, b = −159 ms; Figure 4A). For overall accuracy, a significant interaction was observed (F_2,90_ = 6.476, *p* = 0.002). The separate group's performance deteriorated compared to both the physical (*b* = −6.6%, *p* < 0.001) and concurrent groups (*b* = −5.3%, *p* = 0.010; Figure 4B). The changes in accuracy in the physical and concurrent intervention groups were not different from each other (*p* = 0.487).

#### 3.2.3 Stroop

There was no interaction between group and time for mean reaction time on the Stroop task (F_2,84_ = 0.346, *p* = 0.709). There was, however, a main effect for time (F_1,85_ = 74.987, *p* < 0.001) with all groups improving from pre- to post-intervention (*b* = −159 ms, Figure 4C). For overall accuracy, a significant interaction was observed (F_2,91_ = 3.722, *p* = 0.028). Accuracy within the separate group declined compared to both the physical (*b* = −2.0%, *p* = 0.025) and concurrent groups (*b* = −2.3%, *p* = 0.015; Figure 4D).

#### 3.2.4 Category switch task

##### 3.2.4.1 Switch trials

There was no interaction between group and time (F_2,85_ = 0.203, *p* = 0.817) for reaction time in the switch trials from pre- to post-intervention. There was an effect for time (F_1,87_ = 139.741, *p* < 0.001) such that all groups improved reaction time from pre- to post-intervention (*b* = −355 ms; Figure 4E). For accuracy within the switch trials, there was an interaction between group and time (F_2,91_ = 8.162, *p* < 0.001). This interaction was present since the decrement in accuracy from pre- to post-intervention in the separate group was greater than both the physical (*b* = −6.7%, *p* < 0.001) and concurrent groups (*b* = −4.6%, *p* = 0.011; Figure 4F).

##### 3.2.4.2 Non-switch trials

There was no interaction between group and time (F_2,87_ = 3.699, *p* = 0.900) for reaction time in the non-switch trials. There was an effect for time (F_1,88_ = 102.193, *p* < 0.001) with improvements in reaction time across all groups from pre- to post-intervention (*b* = −246 ms; Figure 4G). For accuracy within the non-switch trials, there was an interaction between group and time (F_2,94_ = 6.932, *p* = 0.002). Examination of the interaction revealed that the decrement in accuracy within the separate group was greater than in both the physical (*b* = 5.8%, *p* < 0.001) and concurrent groups (*b* = 4.6%, *p* = 0.007; Figure 4H).

#### 3.2.5 Millisecond gambling task

The Millisecond Gambling tasks did not reveal any significant results, with no significant interaction (F_2,96_ = 0.176, *p* = 0.839), or main effects for time (F_1,97_ = 0.091, *p* = 0.764; see Appendix Table A5).

#### 3.2.6 Psychomotor vigilance task

There was an interaction between group and time (F_2,89_ = 3.668, *p* = 0.029) within the reaction time data. Reaction time in the physical group deteriorated (slowed) from pre- to post-intervention more than both the separate (*b* = 25 ms, *p* = 0.022) and concurrent groups (*b* = 24 ms, *p* = 0.028; Figure 4I). This was accompanied by an interaction between group and time (F_2,93_ = 4.259, *p* = 0.017) for the number of lapses recorded. A greater increase in the number of lapses from pre- to post-intervention was seen in the physical group compared to both the separate (*b* = 1.2 lapses, *p* = 0.020) and concurrent groups (*b* = 1.2 lapses, *p* = 0.012; Figure 4J).

### 3.3 Impact of interventions on subjective workload (NASA-TLX) after the cognitive battery

#### 3.3.1 Mental load

There was no interaction between group and time (F_2,91_ = 2.479, *p* = 0.089) but there was an effect for time (F_1,92_ = 5.567, *p* = 0.020). Perceived mental load decreased from pre- to post-intervention across all groups (*b* = 6). No other subscale reported a significant interaction effect (other subscale results are reported in the Appendix, see also Supplementary Table A7).

### 3.4 Impact of interventions on physical performance

**Figure 5:**
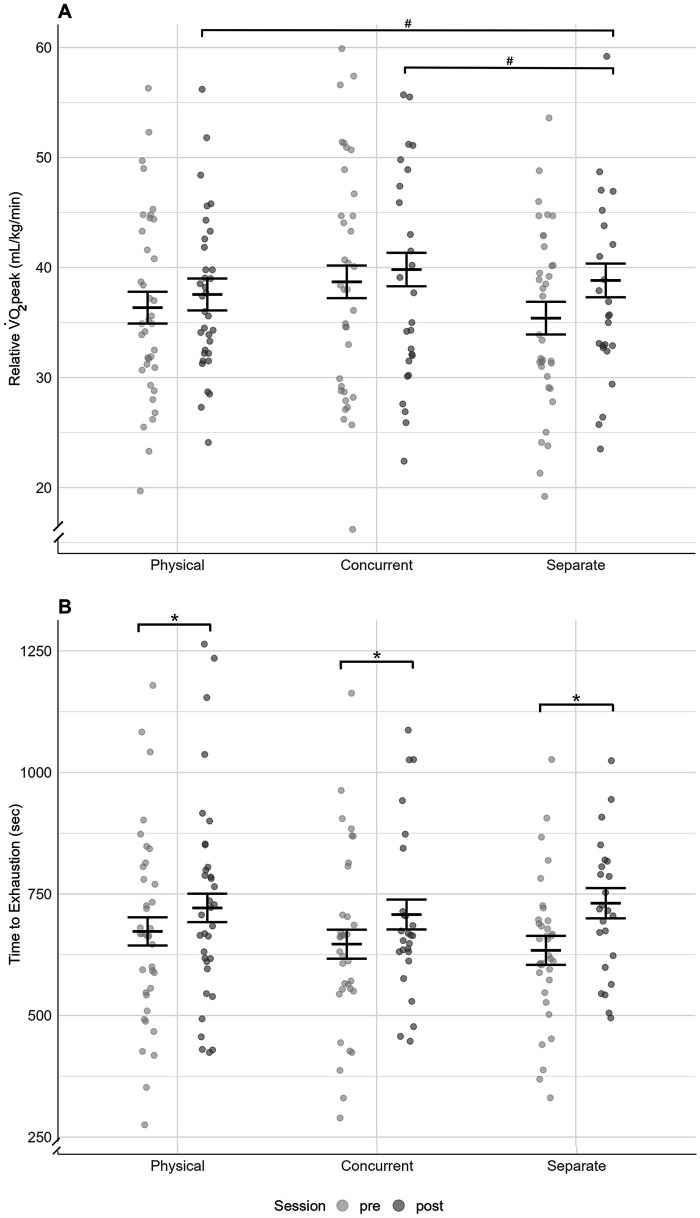
Physical performance outcomes from pre- to post-intervention are presented as boxplots. A: Relative V̇O_2peak_ (mL.kg^−1^.min^−1^); B: Time to exhaustion (seconds). Significant main effects for time (*) and group by time interactions (^#^) are denoted with all effects significant at *p* < 0.05.

There was an interaction between group and time (F_2,82_ = 3.769, *p* = 0.027) such that relative V̇O_2peak_ increased more from pre- to post-intervention in the separate group than both the physical (*b* = 2.2 mL.kg^−1^.min^−1^, *p* *=* 0.017) and concurrent groups (*b* = 2.3 mL.kg^−1^.min^−1^, *p* = 0.019; Figure 5A). There was no interaction for the time-to-exhaustion measure (F_2,82_ = 2.860, *p* = 0.063), but there was an effect for time (F_1,84_ = 57.935, *p* < 0.001) with time-to-exhaustion increasing for all groups from pre- to post-intervention (*b* *=* 66 s; Figure 5B).

## 4 Discussion

This study examined the efficacy of combined physical and cognitive training interventions compared to a physical activity control for improving physical and cognitive outcomes in young, healthy and physically active adults, providing a novel investigation of concurrent training in this population. All intervention groups showed improvements in aspects of physical and cognitive performance. However, differences between the interventions should be considered before implementing this type of training in practice. For example, while reaction time improved across most cognitive assessments, at least partly likely due to learning effects, there were trade-offs with performance accuracy. These trade-offs, however, were not equal across groups, as accuracy was compromised to a greater extent in the separate group. In the psychomotor vigilance task, group differences were also evident. Performance declined in the physical training group, likely reflecting a typical response to repeated testing and the late placement of the PVT within the battery, where reduced motivation, inattention, or boredom may emerge (55). This decrement was less apparent in the groups that received a cognitive training component, suggesting that prior exposure to cognitively demanding tasks may have mitigated these effects through altered mental demand or sustained engagement. Physical performance, assessed by time-to-exhaustion, improved across all groups, with no intervention more effective than another, despite aerobic fitness (relative V̇O_2peak_) improving more in the separate training group. Taken together, these results highlight subtle but meaningful differences in how each intervention influenced physical and cognitive outcomes.

Adherence to the intervention was high (94 ± 7%), with participants required to attend a minimum of 80% of sessions. Given the high adherence, we believe it is unlikely that attendance meaningfully influenced performance outcomes, although we acknowledge that the relationship between adherence and study outcomes was not directly assessed. That being said, the requirement to attend at least 80% of sessions to receive financial reimbursement may have contributed to the drop-out rate. The higher drop-out rate observed in the separate group, compared to the physical training group, may have implications for the broader implementation of separate physical and cognitive training. Additionally, the fact that the concurrent group achieved comparable outcomes despite the halved training time also highlights its potential for more practical and efficient application. Overall, these findings suggest that concurrent physical and cognitive training shows promise as a time-efficient training modality when cognitive improvements are sought alongside physical adaptations.

Across the interventions, response speed improved in tasks requiring rapid executive function (i.e., Visual Search, Stroop, Category Switch), however, in the separate group, these improvements came at a greater cost to accuracy. While accuracy remained relatively stable in both the physical and concurrent groups, significant decreases in accuracy were observed in the separate group (Figure 4). Previous studies involving elite sporting populations reported no compromise to accuracy alongside improvements to reaction time in a Stroop task following separate or sequential interventions (27, 56). There are several methodological differences between those studies and the current one, including the number and activity level of participants, and the timing of the physical and cognitive training, but these factors do not fully account for the differences observed within the present study. Anecdotally, many participants recruited were attracted to this study due to the physical training component, meaning participants in the separate group may have prioritized engagement in the physical training, and assessments, rather than the cognitive component. The nature of the concurrent training, however, provided the potential to engage more holistically with the training, including the cognitive component, which could translate to greater cognitive gains. If this is true, however, the benefits were likely relatively small, or not assessed effectively, as the concurrent group, on occasions, were no better than the physical-only training group.

Although a general slowing of reaction time in the psychomotor vigilance task was observed from pre- to post-intervention across all groups, differences emerged between the physical group and those who underwent cognitive training. Specifically, the physical group showed a greater decline in performance, with slower reaction time and more errors, compared to both the concurrent and separate groups. The overall decline in performance over the 12 weeks may reflect assessment fatigue as participants completed the cognitive battery multiple times during the intervention. Further, greater sensitivity to cognitive performance decrements have been observed when task demands are low (57, 58), consistent with the effort regulation hypothesis (59). The need for active performance regulation is less apparent under these conditions, and performance often declines if sufficient effort is not invested. In this study, the cognitive components of the training may compensate for this, as the inherent engagement of cognitive processes in these interventions may have elicited adaptations related to cognitive control and attention that could support maintained vigilance. Overall, cognitive performance improvements were greatest in the concurrent group, which, when combined with the time efficiency of this approach, provides support for the use of concurrent training. Future research should explore not only its potential cognitive control benefits but also include translational studies and more detailed neurocognitive and neuroimaging research to strengthen our understanding of the underlying mechanisms.

The physical component of the interventions in this study consisted of moderate intensity cycling, and as such, was not designed to optimize physical performance, but rather to contribute to overall physical health and potential neurocognitive adaptations. Therefore, substantial improvements in physical performance were not expected. Nevertheless, an overall improvement in V̇O_2peak_ was observed, with the largest improvements seen in the separate training group. No difference in pre to post V̇O_2peak_ were found between the physical and concurrent training groups. It is unclear why the separate group demonstrated the greatest improvement in this outcome, and we are unable to offer a definitive mechanism to explain this result. One possibility is that by having two distinct training sessions, participants in the separate group had the opportunity to prioritize the physical components. Previous research has indicated that concurrent training paradigms may reduce the relative potency of the individual physical stimulus due to the additional physiological response and attentional demands associated with simultaneous cognitive activity (60). However, this was not evident in the present study, as no differences in power output were observed between the groups (38). Further, physical performance, as assessed by time-to-exhaustion, improved across all groups, with no intervention more effective than another.

Another potential explanation for the disparate physical results could relate to sample characteristics. The higher dropout rate observed in the separate group may have introduced a sample bias, leaving a cohort of more highly motivated and responsive individuals who were willing to commit to the greater time demands of this intervention. This self-selection may have influenced the results in a way that exceeded the capacity of our statistical modelling to fully account for. Further research is needed to investigate these possibilities. However, the higher dropout rate and the greater time investment required for the separate training protocol may also pose practical challenges for broader implementation, limiting its appeal and feasibility in real-world settings.

Despite the greater improvements in V̇O_2peak_ in the separate group, there were no group differences in time-to-exhaustion, with all interventions improving (5.8 to 6.8%) over the 12 weeks. The physical performance improvements for the concurrent group were therefore likely to be independent of cardiorespiratory fitness gains. Indeed, these results align with previous research, albeit from a sequential intervention, which demonstrated improved time-to-exhaustion with no change in V̇O_2peak_ (27). Previous authors suggest that this is due to an attenuated perception of effort (61), whereby, with concurrent training, participants are able to sustain greater physical workloads for longer before reaching maximum exertion, due to an improved tolerance of effort rather than physical adaptations (27, 61). Collectively, these findings highlight the need for further investigation into the mechanisms underpinning performance improvements observed with combined training approaches.

### 4.1 Limitations and future directions

Compared to separate and physical-only training, this double-blinded RCT demonstrates the potential benefits of concurrent physical and cognitive training on elements of both physical and cognitive outcomes, however, the study is not without its limitations. All intervention groups included a physical training component, precluding the examination of a cognitive-only group. The inclusion of a passive or wait-list control group could have clarified the extent to which the interventions benefited cognitive performance beyond changes that can be observed with repeat testing. That said, the inclusion of an active control group reflects real-world physical activity recommendations, enhancing the relevance and applicability of our findings. Additionally, a follow-up period could provide insights into the persistence of performance improvements and potential explanatory mechanisms, particularly if one intervention group showed a greater decay in performance than another.

A further limitation relates to differences in intervention structure between groups. Although the total prescribed physical and cognitive training exposure was matched, the separate training group completed this across a greater number of weekly sessions resulting in a higher overall intervention burden. This may have influenced participant experience in ways not directly captured in the present study, including motivation, fatigue, engagement, boredom, convenience. Consequently, differences between groups may reflect not only training modality, but also these additional intervention-related factors.

It should be acknowledged that the cohort consisted primarily of university students. While this may limit the generalizability of the findings to the wider population, it may also have led to the recruitment of participants closer to their cognitive performance ceiling. Despite these considerations, the concurrent training approach shows promise for enhancing cognitive performance in young, healthy, and cognitively engaged adults. However, if improving cardiorespiratory fitness is the primary goal, caution is advised, as the combined approach used in the present study does not prioritize such adaptations.

Finally, given the number of cognitive outcomes assessed, the potential for chance findings due to multiple comparisons should be considered, and results should be interpreted accordingly.

### 4.2 Practical applications and future research

The findings of this study suggest that concurrent training may be a time-efficient method for training physical and cognitive performance in young and healthy adults. By integrating physical and cognitive components within a single session, concurrent training eliminates the additional time demands, and potential fatigue, associated with training these aspects separately. Further, the overall withdrawal rate in our study (21%) is consistent with prior research on simultaneous physical and cognitive interventions in older adults, which report rates between 12% and 20% (38, 50). The current study observed high withdrawal rates in the separate group (*n* = 11), of which 81% reported to have withdrawn due to the time commitment. This aligns with similar studies that included a separate physical and cognitive training group (62, 63). Intervention acceptability is further explored in McDicken et al. (38). This makes it a practical and effective approach for individuals or populations where both physical and cognitive capabilities are essential.

The approach utilized in this study was informed by a mechanistic rationale suggesting that physical activity could aid synergistic cognitive adaptations. Future research should aim to identify mechanisms and biomarkers that can explain these effects, which would support the development of a precision medicine approach. This would allow for more accurate prescribing of interventions and better prediction of individual responses to training.

## 5 Conclusion

Utilizing individualized and adaptive approaches designed to optimize the training stimulus and grounded in mechanistic rationale, this study suggests that 12 weeks of concurrent physical and cognitive training may support both physical performance and selected aspects of cognitive performance in young, healthy, physically active adults, compared with separate physical and cognitive training or physical training alone. Overall, improvements in physical performance and selected cognitive outcomes were observed, with concurrent training producing outcomes that were at least comparable to, and in some cases better than, both the physical-only and separate physical and cognitive training interventions. The time-efficient nature of combining physical and cognitive training into a single, integrated stimulus may enhance its practical feasibility for broader application and reduce the attendance burden associated with a more time-intensive protocol. While the success of this approach in a young adult population is encouraging, further research is required to explore the underlying mechanisms, refine the protocol, and assess its applicability and translation in other populations and settings.

## Data Availability

The raw data supporting the conclusions of this article will be made available by the authors, without undue reservation.
