# Supplementary material for: Better together - efficacy of combined physical and cognitive training in young adults for enhancing physical and cognitive performance: a randomized controlled trial
Source: Front Sports Act Living. 2026 Jun 24;8:1842483. doi: 10.3389/fspor.2026.1842483 (PMC13341604; doi:10.3389/fspor.2026.1842483)
Supplement: Supplementary file 1 [file Supplementaryfile1.docx]

# Supplementary Material

# Appendix A. Detailed methodology and results

- 1. **Cognitive Battery**

The cognitive battery was completed on a laptop using the keyboard and a USB mouse, linked together in Inquisit player 6 (1) using the ‘batch’ command, allowing participants to seamlessly move from one task to the next, without being interrupted by the researcher. The volume was left up for the reading of digits during the Digit span and the sound effects in the gambling task. No other tasks in the battery contained sound. The whole cognitive battery took about 35 minutes to complete, and the order of tasks was as described above and remained the same for every participant and every assessment.

Auditory Digit Span: Research has highlighted increased retention of facts when physical activity follows a classroom lesson (2). To explore if this relationship is strengthened by concurrent training, the Auditory Digit span task was included to evaluate verbal short-term memory by audibly presenting digit sequences for participants to recall. Participants first completed practice trials to reduce familiarization effects. Participants listened to three (3) numbers in a sequence (“level” 3) and were required to correctly recall the numbers, and sequence, by using the mouse to select the numbers as presented on a number wheel on the screen. Instructions were presented on the screen, and then a minimum of two and maximum of eight practice trials were offered until a correct response was entered. Participants were informed when the practice trial was over and were informed when the test trial was beginning. The task utilized 14 trials starting at level 3 (three digits read out in sequence), after which participants were presented with a wheel of digits on the screen and used the mouse to select their sequence response. Corrections were possible before a submit button was utilized, and the next digit sequence was presented. Digit sequences adjusted to reflect participant performance following a 1:2 staircase. That is, if the response was correct (in digits and sequence order), the participant progressed to the next level (one extra digit in the sequence) while two consecutive incorrect responses would prompt a regression in level (sequence reduced by one digit) (3). After 14 trials of the forward span task, participants would see a new page of instructions for the backward span task. During the backward trials, participants were required to recall the digits presented in reverse order. The backward task started at level 2 (two digits presented in sequence). Only single digits (i.e., 3, 7, etc.; not 24, 42, etc.) were presented for both forward and backward span tasks and the overall assessment was four minutes in duration (1). Two-error maximum length (TE_ML) was used as a traditional measure of digit span performance. This measures the correct digit span a participant achieves before making two consecutive errors and regressing to a lower level.

Table A1. Descriptive data for Digit Span Forward and Backward trials variables by group and time point.

| Outcome | Intervention |  | Pre |  | Mid |  | Post |  |
| --- | --- | --- | --- | --- | --- | --- | --- | --- |
|  |  |  | Mean (SD) |  | Mean (SD) |  | Mean (SD) |  |
| fTE_ML (n)  Forward Two-Error Maximum Length | Physical |  | 6.4 (1.4) |  | 6.8 (1.4) |  | 6.8 (1.5) |  |
|  | Concurrent |  | 6.4 (1.2) |  | 7.0 (1.1) |  | 7.2 (1.6) |  |
|  | Separate |  | 6.6 (1.3) |  | 7.4 (1.3) |  | 7.0 (1.5) |  |
| bTE_ML (n)  Backward Two-Error Maximum Length | Physical |  | 5.9 (1.4) |  | 6.1 (1.6) |  | 6.7 (1.5) |  |
|  | Concurrent |  | 6.0 (1.5) |  | 6.4 (1.6) |  | 6.6 (1.6) |  |
|  | Separate |  | 5.9 (1.6) |  | 6.1 (1.5) |  | 6.9 (1.5) |  |

Abbreviation: SD = Standard Deviation

- Inquisit Visual search Task (Divided Attention): This task measured visuo-spatial working memory by having participants signal the presence or absence of a target stimuli (red or green, Ts or Ls randomly rotated in increments of 60 degrees) by pressing one of two buttons. Each trial commenced with the initial fixation on a small dot centered on the screen. Participants indicate their readiness to progress by pressing a key on the keyboard, changing the screen to a brief display of the target stimuli for that trial. Shortly after the display of the target (1000ms), the screen would change to show an array of distractor elements, of which the target stimuli were amongst. The distractors were an assortment of elements, each sharing one common feature with the target, i.e. the same letter or color. Participants were required to visually scan the array of elements on the screen to determine whether their target (matching color, letter, and orientation) was present. Responses of either present or absent were indicated using allocated keys ‘E’ and ‘I’ respectively. The button press terminated the display and progressed the participant to the next trial unless the response took longer than 7000ms, in which case the trial would terminate and the next would automatically commence.

The array of elements contained either 6, 12, 24, or 48 distractor stimuli along with the single target element. The allocation of target types, distractor types and the number of distractors for each trial was randomized and was not dictated by correct or incorrect responses. The visual search task was about four minutes, consisting of eight practice trials and 48 test trials. Given participants were instructed to respond as quickly and accurately as possible, speed and accuracy were deemed to be of equal importance. Mean reaction time (meanRT) was calculated from target detection responses filtered to exclude incorrect detections. Overall accuracy (ACCoverall) was presented as a proportion of correct responses across all trials (1).

Results of the mid-intervention assessment for the Visual Search task.

For the Visual Search Task, data from the mid-intervention assessment (Appendix, Table A2) were included in the analysis to further explore the significant interaction observed from pre- to post-intervention in the accuracy measure. There was a main effect for time (F_1,94_=67.869, *p*<0.001) such that the overall reaction time was faster across all groups at the mid-point compared to pre-intervention (*b*=265 ms). This was accompanied by an interaction between group and time for accuracy from pre- to mid-intervention (F_2,97_=3.585, *p*=0.031). Pre- to mid-intervention improvements in accuracy in the physical group were significantly greater than the changes observed in both the separate (*b*=4.6%, *p*=0.018) and concurrent groups (*b*=4.1%, *p*=0.034). No differences were observed in reaction time between mid- and post-intervention. There was an interaction between group and time for accuracy between mid- and post-intervention (F_2,85_=4.628, *p*=0.012). In this case, accuracy improved in the concurrent group relative to the separate group (*b*=5.0%, *p*=0.032) from mid- to post-intervention.

Table A2. Descriptive data for the Visual Search task variables by group and time point.

| Outcome | Intervention |  | Pre |  | Mid |  | Post |  |
| --- | --- | --- | --- | --- | --- | --- | --- | --- |
|  |  |  | Mean (SD) |  | Mean (SD) |  | Mean (SD) |  |
| MeanRT (ms)  Mean reaction time | Physical |  | 1964 (508) |  | 1742 (384) |  | 1712 (368) |  |
|  | Concurrent |  | 1896 (390) |  | 1582 (353) |  | 1620 (259) |  |
|  | Separate |  | 1804 (364) |  | 1506 (355) |  | 1470 (380) |  |
| ACCoverall  Accuracy overall  Proportion correct as a % | Physical |  | 89 (10) |  | 92 (4) |  | 91 (5) |  |
|  | Concurrent |  | 91 (6) |  | 90 (6) |  | 92 (4) |  |
|  | Separate |  | 91 (7) |  | 90 (7) |  | 87 (9) |  |

Abbreviation: SD = Standard Deviation

Color Word Stroop with Keyboard Responding: Executive functions such as response inhibition are key components of concentration and are potential targets for concurrent training protocols. Response inhibition is the ability to concentrate on relevant information to make an appropriate response, even when distracting information is present. This task implements a version of the traditional Stroop Task (4) utilizing keyboard input which demonstrates the interference caused by the word’s meaning on the identification of the color in which the words are presented. This is based on both reaction time and accuracy differences to both congruent (color of the text matches the meaning of the word) and incongruent (color of the text does not match the meaning of the word) combinations. The control condition consisted of colored rectangles.

The Stroop consisted of four words (blue, green, red, and black) serially displayed in the center of a computer screen. The words remain displayed until participants respond by pressing one of four letters on a keyboard that correspond to the color the word was written in, not the meaning of the word. Participants were provided with the following keyboard response options - D (red), F (green), J (blue), and K (black). For example, if the word “blue” is written in red coloring, the correct response would be the letter D to indicate red. Participants were instructed to respond as quickly and accurately as possible. The assessment consisted of 108 trials (36 of each condition) with an intertrial interval of 200ms making it approximately three to four minutes long. The trial stimuli were randomly sampled.

The Stroop task provided several outcomes related to accuracy and RT to different trial types. Overall, Stroop accuracy (propcorrect) was reported as the overall proportion of all trials that were correct. Of these correct trials (filtered to exclude incorrect trials), the overall mean latency (meanRT in ms) was also recorded.

Results of the mid-intervention assessment for the Stroop task.

Data from the mid-intervention assessment (Appendix, Table A3) for the Stroop task were included in the analysis to explore further the significant interaction in Stroop results observed from pre-to post-intervention For reaction time, there was no interaction (F_2,92_=0.843, *p*=0.434) but there was a main effect for time (F_1,94_=91.156, *p*<0.001) with reaction time improving across all groups from pre- to mid-assessment (*b*=137 ms faster). This improvement in reaction time was however, accompanied by an effect for time (F_1,100_=13.483, *p*<0.001) within the accuracy results, with a reduction in accuracy across all groups (*b*=-1.5%). There was no group x time interaction effect for accuracy (F_2,98_=1.801, *p*=0.171).

From the mid-assessment to the post-assessment, there was no interaction between group and time for the reaction time measure (F_2,81_=0.137, *p*=0.872), but there was an effect for time (F_1,83_=4.658, *p*=0.034) such that reaction time improved further across all groups (*b*=24 ms). There was no significant interaction (F_2,83_=2.007, *p*=0.141), nor main effect for time (F_1,85_=3.393, *p*=0.069) for accuracy between mid- and post-intervention.

Table A3. Descriptive data for Stroop variables by group and time point.

| Outcome | Intervention |  | Pre |  | Mid |  | Post |  |
| --- | --- | --- | --- | --- | --- | --- | --- | --- |
|  |  |  | Mean (SD) |  | Mean (SD) |  | Mean (SD) |  |
| meanRT (ms)  Mean reaction time | Physical |  | 966 (312) |  | 860 (232) |  | 833 (190) |  |
|  | Concurrent |  | 973 (217) |  | 811 (181) |  | 777 (148) |  |
|  | Separate |  | 939 (295) |  | 754 (191) |  | 722 (142) |  |
| Overall Accuracy Proportion correct as a % | Physical |  | 98 (2) |  | 97 (2) |  | 96 (3) |  |
|  | Concurrent |  | 97 (2) |  | 96 (3) |  | 96 (3) |  |
|  | Separate |  | 97 (3) |  | 94 (6) |  | 93 (5) |  |

Abbreviation: SD = Standard Deviation

Category Switch: Concurrent training by nature necessitates the repeated switching of attention, and consequently remains effortful, thereby providing a stimulus for enhancing one’s ability to control their task switching ability. The task switching paradigm requires that participants switch between two-word categorization tasks. In each trial participants were presented with a word in the center of their computer screen. The words were concrete nouns chosen randomly from a selection of 16 words (adapted from Mayr and Kliegl, 2000) (5) grouped according to four categories; large, nonliving objects (*table, bicycle, coat, TV*), small, nonliving objects (*pebble, knob, cup, marble*), large, living objects (*whale, alligator, shark, lion*) and small, living objects (*mouse, sparrow, goldfish, frog*). Participants were required to define each object as being living/nonliving and larger/smaller than a basketball depending on a symbol that appeared above the word. The symbols were either a heart to indicate living vs. nonliving, and a cross to indicate larger vs. smaller than a basketball. Participants were instructed to answer as quickly and accurately as possible using two allocated keys on the keyboard (‘E’ for living/larger and ‘I’ for nonliving/smaller). Half of the trials were switch trials between congruent (correct responses for both categories require the same button to be pressed) and incongruent (correct responses for both categories require opposite buttons to be pressed). Some modifications were made to the default Millisecond script to limit ambiguity of words (i.e. ‘lizard’ was changed to ‘frog’ due to the variety of lizard sizes) and to condense the duration of the assessment. The number of practice trials for each condition was reduced from 32 to 8; the number of combined practice trials was kept at 16. The number of trials in the test was reduced from 84 to 32. This ensured that the task took around five minutes for participants to complete (1,5,6).

Results of the mid-intervention assessment for the Category Switch task.

Data from the mid-intervention assessment (Appendix, Table A4) for the Category Switch task were included in the analysis to explore further the significant interaction in Category Switch task results observed from pre-to post-intervention. Within the non-switch trials, reaction time, from pre- to mid-intervention, there was no interaction (F_2,96_=0.253, *p*=0.777). There was, however, an effect for time (F_1,98_=93.510, *p*<0.001) with reaction time improving across all groups from pre- to mid-intervention (*b*=211 ms faster). There was no interaction (F_2,102_=1.612, *p*=0.205) nor effect for time (F_1,104_=1.998, *p*=0.160) within the accuracy results of the non-switch trials from pre- to mid-intervention.

Within the non-switch trials, reaction time, from mid- to post-intervention, there was no interaction (F_2,83_=0.732, *p*=0.484), nor any effect for time (F_1,84_=2.901, *p*=0.092). There was no interaction (F_2,87_=2.360, *p*=0.100) nor effect for time (F_1,88_=0.153, *p*=0.697) within the accuracy results of the non-switch trials from mid- to post-intervention.

Within the switch trials, reaction time, from mid- to post-intervention, there was no interaction (F_2,95_=0.651, *p*=0.524) but there was a main effect for time (F_1,97_=144.443, *p*<0.001) with reaction time improving across all groups from pre- to mid-assessment (*b*=332 ms faster). There was no interaction (F_2,100_=1.731, *p*=0.183) nor effect for time (F_1,102_=0.615, *p*=0.435) within the accuracy results of the switch trials from pre- to mid-intervention.

Within the switch trials, reaction time, from mid- to post-intervention, there was no interaction (F_2,82_=0.200, *p*=0.819) nor any main effect for time (F_1,84_=3.240, *p*=0.075). There was no interaction (F_2,86_=2.363, *p*=0.100) nor an effect for time (F_1,88_=1.708, *p*=0.195) within the accuracy results of the switch trials from mid- to post-intervention.

Table A4. Descriptive data for Category Switch variables by group and time point.

| Outcome | Intervention |  | Pre |  | Mid |  | Post |  |
| --- | --- | --- | --- | --- | --- | --- | --- | --- |
|  |  |  | Mean (SD) |  | Mean (SD) |  | Mean (SD) |  |
| MeanRT_Switch (ms)  Mean latency of switch trials | Physical |  | 1420 (544) |  | 1137 (351) |  | 1103 (358) |  |
|  | Concurrent |  | 1386 (400) |  | 1060 (295) |  | 1042 (387) |  |
|  | Separate |  | 1251 (356) |  | 893 (192) |  | 830 (139) |  |
| MeanRT_nonSwitch (ms)  Mean latency of non-switch trials | Physical |  | 1149 (449) |  | 970 (257) |  | 931 (229) |  |
|  | Concurrent |  | 1125 (301) |  | 904 (208) |  | 895 (287) |  |
|  | Separate |  | 992 (184) |  | 767 (126) |  | 718 (106) |  |
| Accuracy_switch  Proportion of correct switch trials as a % | Physical |  | 93 (7) |  | 93 (6) |  | 94 (6) |  |
|  | Concurrent |  | 92 (8) |  | 92 (5) |  | 92 (5) |  |
|  | Separate |  | 94 (6) |  | 92 (8) |  | 89 (6) |  |
| Accuracy_nonswitch  Proportion of correct non-switch trials as a % | Physical |  | 95 (5) |  | 96 (4) |  | 96 (5) |  |
|  | Concurrent |  | 94 (6) |  | 93 (7) |  | 94 (6) |  |
|  | Separate |  | 96 (4) |  | 94 (4) |  | 92 (7) |  |

Abbreviation: SD = Standard Deviation

Millisecond Gambling: The Millisecond Gambling Task is an adapted version of the Cambridge Gambling Task which assesses decision making and risk-taking behavior. Participants are presented with a screen consisting of ten boxes colored either red or blue. The ratio of red to blue boxes varied for each trial from 1:9 to 9:1. The assessment consisted of three stages; a practice round, one block of nine trials with ascending bets, and one block of nine trials with descending bets. During the practice, of which there were five trials, participants are told (in the instruction screen) that hidden under one of the red or blue boxes is a yellow token. Their task is to choose which color they suspect the token to be under, by selecting either the ‘RED’ or ‘BLUE’ nomination box, positioned at the bottom of the screen. The participants are provided with immediate feedback as to whether they were correct or not. The ascending round introduced a betting system which enabled participants to wager points on their decision. Available bets were displayed in a box positioned on the right-hand side of the screen. Upon selecting their chosen color, a baseline bet would appear in the box and incrementally increase at intervals of 2000 milliseconds (ms) which was reduced from 5000 ms in the default script to reduce the task duration. The bet increments were based on a percentage of the current total of points earned. Five bets were offered per trial, representative of 5%, 25%, 50%, 75% and 95% of current points. Participants commenced the assessment with 100 points and were instructed to try to increase this total as much as they could, but no monetary value was placed on the total points achieved by the task's completion. To choose and submit a bet, the participants were instructed to click on the bet box, which would stop the bets from continuing to incrementally increase. If the participant chose the correct color, they won the number of points submitted. If they chose the incorrect color, the number of points was deducted from their running total. During the descending round, participants were provided with the same instructions, of clicking on the bet box, to submit their chosen number of points to wager however, in this serial the bets would instead begin at the ceiling 95%-point bet and incrementally decrease at intervals of 2000 ms. The assessment duration was approximately four minutes (7,8).

Table A5. Descriptive data for Millisecond Gambling variables by group and time point.

| Outcome | Intervention |  | Pre |  | Mid |  | Post |  |
| --- | --- | --- | --- | --- | --- | --- | --- | --- |
|  |  |  | Mean (SD) |  | Mean (SD) |  | Mean (SD) |  |
| Best choice bet (%) | Physical |  | 97 (7) |  | 97 (5) |  | 96 (5) |  |
|  | Concurrent |  | 96 (9) |  | 97 (4) |  | 96 (7) |  |
|  | Separate |  | 97 (5) |  | 96 (7) |  | 97 (6) |  |

Abbreviation: SD = Standard Deviation

Psychomotor vigilance task (PVT): Performance on tasks requiring sustained attention has been shown to be sensitive to interventions that integrate challenging cognitive tasks with physical activity (9,10). The script used reflected a simple motor reaction time test widely used to measure vigilant and sustained attention (11). Participants were instructed to press the spacebar key on a keyboard as soon as possible upon seeing a red stopwatch appear in the center of their computer screen. Participants were provided with immediate feedback displaying their reaction time for valid responses (pressing the key after the stopwatch has appeared). An error message was displayed for invalid responses defined as being either too soon (i.e. <100ms) or before the stopwatch had appeared (i.e. false start). Stimuli appeared in a random pattern with an inter-stimulus interval varying between 2000 and 10,000 milliseconds. The script duration was reduced from 10 minutes to five minutes as per [Loh](https://pubmed.ncbi.nlm.nih.gov/15354700/) et al. (12) which suggests that 5 minutes may be sufficient time, especially late in the test battery. Measures of sustained attention during the PVT selected for analysis were mean response latency (including responses over 500ms) (meanRT), and number of lapses (NumberOfLapses), indicating an inability to perform a timely response (13).

Results of the mid-intervention assessment for the Psychomotor Vigilance Task.

Data from the mid-intervention assessment (Appendix, Table A6) for the Psychomotor Vigilance Task were included in the analysis to explore further the significant interaction in Psychomotor Vigilance Task results observed from pre-to post-intervention. From pre- to mid-intervention, there was no interaction between group and time (F_2,98_=1.425, *p*=0.245) within the mean reaction time data. There was an effect for time (F_1,100_=4.683, *p*=0.033) with all groups increasing (slowing) their reaction time from pre- to mid-intervention (*b*=26 ms). A similar result was observed in the number of lapses. There was no interaction (F_2,95_=2.176, *p*=0.119), but there was a main effect for time (F_1,97_=7.515, *p*=0.007) with the number of lapses increasing (*b*=0.5 lapses, *p*=0.007) from pre- to mid-intervention across all groups.

From the mid-assessment to the post-assessment, there was no interaction between group and time for mean reaction time (F_2,87_=0.268, *p*=0.765) and no main effect for time (F_1,89_=0.623, *p*=0.432). Similarly, for the change in the number of lapses from mid- to post-assessment, there was no interaction (F_2,84_=0.711, *p*=0.494) and no main effect for time (F_1,85_=1.930, *p*=0.168).

Table A6. Descriptive data for PVT variables by group and time point.

| Outcome | Intervention |  | Pre |  | Mid |  | Post |  |
| --- | --- | --- | --- | --- | --- | --- | --- | --- |
|  |  |  | Mean (SD) |  | Mean (SD) |  | Mean (SD) |  |
| MeanRT (ms)  Mean reaction time | Physical |  | 306 (29) |  | 359 (191) |  | 338 (60) |  |
|  | Concurrent |  | 306 (32) |  | 314 (39) |  | 313 (36) |  |
|  | Separate |  | 316 (39) |  | 329 (62) |  | 322 (29) |  |
| Number Of Lapses (n) | Physical |  | 0 (1) |  | 1 (2) |  | 2 (3) |  |
|  | Concurrent |  | 1 (1) |  | 1 (2) |  | 1 (1) |  |
|  | Separate |  | 1 (1) |  | 1 (1) |  | 1 (1) |  |

Abbreviation: SD = Standard Deviation

**NASA-Task Load Index**

To assess subjective load, the NASA-Task Load Index (TLX) survey was completed following the performance of the cognitive battery at pre- and post-intervention. The NASA TLX is a multi-dimensional rating procedure which uses six subscales to quantify overall workload, five of which were used in the present study: mental demand, physical demand, temporal demand, effort, and frustration. Participants were instructed to rate their perceived load for the entire cognitive battery according to each of the subscales.

Results of the NASA-Task Load Index subscales

***Mental load*:** There was no interaction between group and time (F_2,91_=2.479, *p*=0.089) but there was an effect for time (F_1,92_=5.567, *p*=0.020). Perceived mental load decreased from pre- to post-intervention across all groups (*b*=6).

***Physical load:*** There was no interaction between group and time (F_2,88_=0.702, *p*=0.499), but there was an effect for time (F_1,89_=4.308, *p*=0.041). Perceived physical load decreased from pre- to post-intervention across all groups (*b*=4).

***Temporal load*:** There was no interaction between group and time (F_2,91_=3.050, *p*=0.052), with no effect for time (F_1,92_=3.242, *p*=0.075) for the temporal load subscale.

***Effort*:** There was no interaction between group and time (F_2,88_=1.416, *p*=0.248) but there was an effect for time (F_1,89_=13.550, *p*<0.001). Perceived effort decreased from pre- to post-intervention across all groups (*b*=8).

***Frustration*:** There was no interaction between group and time (F_2,93_=1.577, *p*=0.212), but there was an effect for time (F_1,94_=4.455, *p*=0.037). Frustration decreased from pre- to post-intervention across all groups (*b*=6).

Table A7. Descriptive data for NASA-TLX variables by group and time point.

| Outcome | Intervention |  | Pre |  | Mid |  | Post |  |
| --- | --- | --- | --- | --- | --- | --- | --- | --- |
|  |  |  | Mean (SD) |  | Mean (SD) |  | Mean (SD) |  |
| Mental load | Physical |  | 64 (24) |  | 59 (24) |  | 56 (24) |  |
|  | Concurrent |  | 66 (16) |  | 44 (24) |  | 54 (22) |  |
|  | Separate |  | 61 (26) |  | 54 (24) |  | 65 (26) |  |
| Physical load | Physical |  | 18 (18) |  | 16 (18) |  | 0 (17) |  |
|  | Concurrent |  | 20 (17) |  | 17 (18) |  | 14 (12) |  |
|  | Separate |  | 18 (20) |  | 13 (15) |  | 18 (21) |  |
| Temporal load | Physical |  | 52 (22) |  | 49 (24) |  | 44 (27) |  |
|  | Concurrent |  | 57 (19) |  | 43 (22) |  | 45 (24) |  |
|  | Separate |  | 50 (25) |  | 47 (28) |  | 57 (25) |  |
| Effort | Physical |  | 62 (22) |  | 58 (22) |  | 57 (21) |  |
|  | Concurrent |  | 65 (21) |  | 51 (24) |  | 50 (24) |  |
|  | Separate |  | 65 (16) |  | 52 (26) |  | 63 (23) |  |
| Frustration | Physical |  | 39 (22) |  | 32 (22) |  | 31 (19) |  |
|  | Concurrent |  | 41 (25) |  | 27 (25) |  | 27 (19) |  |
|  | Separate |  | 37 (27) |  | 24 (25) |  | 38 (30) |  |

Abbreviation: SD = Standard Deviation

## Maximal incremental fitness assessment:

This approach seeks to not only evaluate intervention effects of physical performance but attempts to understand what might underly any changes. Given the intervention requirement for physical training, it is important to evaluate whether the different interventions altered adaptations that may impact physical performance. This could have occurred, for example through adaptations to effort-based pathways or in the different heart rates evoked for a given load during the concurrent vs physical training.

Peak oxygen uptake (absolute and relative): Upon arrival, and after completing the cognitive battery, each participant had their height and weight recorded and was fitted with a heart rate monitor (Polar H10). To assess V̇O_2peak_, each participant completed an incremental maximal fitness test on an upright stationary cycle ergometer (Lode Corvial CPET, Lode, Netherlands), with respiratory data collected via a metabolic cart (Vyntus CPX, Vyaire Medical, America). It was reiterated that participants needed to continue in the assessment for as long as they could to ensure an accurate measure of fitness was obtained. The assessment protocol included a three-minute warm up at 50 W followed by an incremental assessment period where the resistance on the bike increased every two minutes by 25 W for females or 50 W for males. Participants were required to pedal at a cadence between 60 and 80 revolutions per minute (RPM). HR was recorded via Polar H10 monitor every 15 seconds. The test was terminated when the participant voluntarily stopped, or their RPM fell below 60, and they were unable to increase it within 5 seconds. Once the test was terminated, the participant remained on the bike for a minimum of two minutes allowing the assessor to monitor heart rate recovery.

Cardiorespiratory fitness is a large contributor to aerobic endurance performance and has been associated with performance on tasks requiring cognitive control (14,15). V̇O_2peak_ was recorded through breath-by-breath gas analysis and then reported using a 15-second average giving a V̇O_2peak_ value for every recorded HR.

TTE*:* More recent studies have shown that adding cognitive training (not simultaneously) to physical training sessions can lead to improved endurance capacity during time-to-exhaustion tests (43). Thus, it may increase disproportionately to fitness changes in groups with a cognitive component. Time to exhaustion (TTE) is reflective of fitness but is also representative of the effort level that performers are willing to engage with.

Results of the mid-intervention assessment for relative V̇O_2peak_.

Data from the mid-intervention assessment (Appendix, Table A7) for relative V̇O_2peak_ were included in the analysis to explore further the significant interaction in relative V̇O_2peak_ observed from pre- to post-intervention. From pre- to mid-intervention, there was no interaction between group and time (F_2,92_=0.006, *p*=0.994), but there was an effect for time (F_1,94_=16.186, *p*<0.001) with all groups increasing their relative V̇O_2peak_ from pre- to mid-intervention (*b*=1.4 mL.kg^-1^.min^-1^).

From the mid-assessment to the post-assessment, there was no significant interaction between group and time (F_2,82_=2.540, *p*=0.085) and no main effect for time (F_1,84_=0.254 *p*=0.616).

Table A8. Descriptive data for physical performance variables by group and time point.

| Outcome | Intervention |  | Pre |  | Mid |  | Post |  |
| --- | --- | --- | --- | --- | --- | --- | --- | --- |
|  |  |  | Mean (SD) |  | Mean (SD) |  | Mean (SD) |  |
| Relative V̇O_2peak_  (mL.kg.min) | Physical |  | 36.4 (8.5) |  | 37.6 (7.6) |  | 37.4 (7.1) |  |
|  | Concurrent |  | 38.7 (10.6) |  | 39.4 (10.1) |  | 38.5 (9.7) |  |
|  | Separate |  | 35.4 (8.2) |  | 36.3 (8.6) |  | 37.3 (8.3) |  |
| Absolute V̇O_2peak_  (mL.min) | Physical |  | 2639 (815) |  | 2744 (764) |  | 2712 (734) |  |
|  | Concurrent |  | 2626 (890) |  | 2624 (846) |  | 2610 (901) |  |
|  | Separate |  | 2539 (779) |  | 2605 (857) |  | 2715 (895) |  |
| Respiratory Exchange Ratio | Physical |  | 1.2 (0.1) |  | 1.2 (0.1) |  | 1.2 (0.1) |  |
|  | Concurrent |  | 1.2 (0.1) |  | 1.2 (0.1) |  | 1.3 (0.1) |  |
|  | Separate |  | 1.2 (0.1) |  | 1.2 (0.1) |  | 1.3 (0.1) |  |
| HR peak (bpm) | Physical |  | 181.1 (12.1) |  | 179.5 (14.2) |  | 181.4 (12.8) |  |
|  | Concurrent |  | 179.4 (13.8) |  | 180.3 (10.8) |  | 179.7 (17.8) |  |
|  | Separate |  | 180.0 (12.9) |  | 178.3 (18.5) |  | 184.8 (10.2) |  |
| Time to exhaustion  (seconds) | Physical |  | 673 (196) |  | 719 (193) |  | 732 (212) |  |
|  | Concurrent |  | 648 (186) |  | 690 (184) |  | 701 (170) |  |
|  | Separate |  | 634 (145) |  | 671 (137) |  | 720 (139) |  |

Abbreviation: SD = Standard Deviation

# Appendix B. Withdrawals

Table B1: Reasons for withdrawing from the study. Participants were asked if they were willing to provide a reason upon withdrawal. Participants who stopped attending training sessions or responding to communication from the research team were marked as “stopped attending training sessions”.

| Reason for withdrawing | Physical | Concurrent | Separate | Not assigned to a group | Total |
| --- | --- | --- | --- | --- | --- |
| Time commitment | 1 | 7 | 9 | 1 | 18 |
| Stopped attending training sessions | 2 | 1 | 1 |  | 4 |
| Illness |  | 1 | 1 |  | 2 |
| Injury |  |  |  | 2 | 2 |
| Total | 3 | 9 | 11 | 3 | 26 |

# References

1. Inquisit 6. Millisecond [Internet]. 2015 [cited 2024 Nov 22]. Available from: <https://www.millisecond.com/>. (Accessed: November 11, 2024)

2. van Dongen EV, Kersten IH, Wagner IC, Morris RG, Fernández G. Physical exercise performed four hours after learning improves memory retention and increases hippocampal pattern similarity during retrieval. Curr Biol. 2016;26:1722–1727.

3. Woods DL, Kishiyamaa MM, Lund EW, Herron TJ, Edwards B, Poliva O, Hink RF, Reed B. Improving digit span assessment of short-term verbal memory. J Clin Exp Neuropsychol. 2011;33:101–111. doi: 10.1080/13803395.2010.493149.

4. Stroop JR. Studies of interference in serial verbal reactions. J Exp Psychol. 1935;18:643.

5. Mayr U, Kliegl R. Task-set switching and long-term memory retrieval. J Exp Psychol Learn Mem Cogn. 2000;26:1124–1140.

6. Muhmenthaler MC, Meier B. Different impact of task switching and response-category conflict on subsequent memory. Psychol Res. 2021;85:679–696. doi: 10.1007/s00426-019-01274-3.

7. Rogers RD, Everitt BJ, Baldacchino A, Blackshaw AJ, Swainson R, Wynne K, Baker NB, Hunter J, Carthy T, Booker E, et al. Dissociable Deficits in the Decision-Making Cognition of Chronic Amphetamine Abusers, Opiate Abusers, Patients with Focal Damage to Prefrontal Cortex, and Tryptophan-Depleted Normal Volunteers: Evidence for Monoaminergic Mechanisms. Neuropsychopharmacology. 1999;20:322–339. doi: 10.1016/S0893-133X(98)00091-8.

8. Romeu RJ, Haines N, Ahn W-Y, Busemeyer JR, Vassileva J. A computational model of the Cambridge gambling task with applications to substance use disorders. Drug Alcohol Depend. 2020;206:107711. doi: 10.1016/j.drugalcdep.2019.107711.

9. Zhu W, Wadley VG, Howard VJ, Hutto B, Blair SN, Hooker SP. Objectively Measured Physical Activity and Cognitive Function in Older Adults. Med Sci Sports Exerc. 2017;49:47–53. doi: 10.1249/mss.0000000000001079.

10. Maclean KA, Ferrer E, Aichele SR, Bridwell DA, Zanesco AP, Jacobs TL, King BG, Rosenberg EL, Sahdra BK, Shaver PR, et al. Intensive Meditation Training Improves Perceptual Discrimination and Sustained Attention. Psychol Sci. 2010;21:829–839. doi: 10.1177/0956797610371339.

11. Thomann J, Baumann CR, Landolt H-P, Werth E. Psychomotor Vigilance Task Demonstrates Impaired Vigilance in Disorders with Excessive Daytime Sleepiness. J Clin Sleep Med. 2014;10:1019–1024. doi: 10.5664/jcsm.4042.

12. Loh S, Lamond N, Dorrian J, Roach G, Dawson D. The validity of psychomotor vigilance tasks of less than 10-minute duration. Behav Res Methods Instrum Amp Comput. 2004;36:339–346. doi: 10.3758/bf03195580.

13. Sinclair KL, Ponsford JL, Rajaratnam SMW, Anderson C. Sustained attention following traumatic brain injury: Use of the Psychomotor Vigilance Task. J Clin Exp Neuropsychol. 2013;35:210–224. doi: 10.1080/13803395.2012.762340.

14. Pontifex MB, Scudder MR, Drollette ES, Hillman CH. Fit and vigilant: The relationship between poorer aerobic fitness and failures in sustained attention during preadolescence. Neuropsychology. 2012;26:407–413. doi: 10.1037/a0028795.

15. Voss MW, Chaddock L, Kim JS, Vanpatter M, Pontifex MB, Raine LB, Cohen NJ, Hillman CH, Kramer AF. Aerobic fitness is associated with greater efficiency of the network underlying cognitive control in preadolescent children. Neuroscience. 2011;199:166–176. doi: 10.1016/j.neuroscience.2011.10.009.
